# Lack of evidence for an effect of joint line obliquity after medial opening wedge high tibial osteotomy: A 12‐year single‐centre retrospective study

**DOI:** 10.1002/jeo2.70655

**Published:** 2026-03-25

**Authors:** Lilia Gharbi, Jean‐François Gonzalez, Michael Lopez, Joseph Attas, Nicolas Bronsard, Régis Bernard de Dompsure, Lolita Micicoi, Grégoire Micicoi

**Affiliations:** ^1^ IULS‐University Institute for Locomotion and Sports, Pasteur 2 Hospital University Côte d'Azur Nice France; ^2^ ICARE Unit, Côte d'Azur University, Inserm, CNRS Valrose Institute of Biology Nice Provence‐Alpes‐Côte d'Azur France

**Keywords:** functional outcomes, high tibial osteotomy, joint line obliquity, long‐term survival, medial opening wedge

## Abstract

**Purpose:**

Large correction in opening wedge high tibial osteotomy (HTO) may lead to excessive joint line obliquity (JLO). This study hypothesized that the presence of an oblique joint line, defined by knee joint line obliquity (KJLO) > 5° and/or medial proximal tibial angle (MPTA) > 94°, was associated with poorer functional outcomes and lower survival rates following HTO at long‐term follow‐up.

**Methods:**

This retrospective study included 83 patients who underwent a medial opening wedge HTO with at least 5 years of follow‐up. Complete pre‐ and postoperative radiographic parameters (hip–knee–ankle angle [HKA], MPTA, lateral distal femoral angle [LDFA], KJLO, joint line convergence angle [JLCA]) and functional outcomes (Knee injury and Osteoarthritis Outcome Score [KOOS], International Knee Society Score [IKS], Subjective Knee Value [SKV]) were analysed. Excessive JLO was defined by a KJLO ≥ 5° and/or an MPTA > 94°. An independent Student's *t* test was then performed to compare functional scores and angular measurements between groups. Functional results and revision‐free survival were analysed according to JLO, using the Kaplan–Meier method for survival analysis. The mean follow‐up was 12.3 ± 3.3 years (5–19).

**Results:**

The HKA, MPTA, JLCA and KJLO angles varied significantly (ΔHKA = 7.8° ± 3.7, *p* < 0.001). Functional scores showed no significant difference between patients with or without JLO. Five‐ and 10‐year survival rates were 86% and 71%, respectively, with no difference based on JLO (*p* = 0.500). Smoking, age > 55 years, Ahlbäck stage III osteoarthritis and out‐of‐target postoperative alignment (HKA < 180° or > 183°) were identified as risk factors for conversion to total knee arthroplasty in multivariate Cox regression analysis.

**Conclusion:**

This retrospective analysis found no significant association between excessive JLO (KJLO ≥ 5° and/or MPTA > 94°) and either survival or functional outcomes after medial opening wedge HTO at a mean follow‐up of 12 years. Factors negatively influencing survival after HTO were smoking, age > 55 years, advanced osteoarthritis and out‐of‐target postoperative alignment. These findings should be confirmed by larger series.

**Level of Evidence:**

Level III.

## Graphical abstract

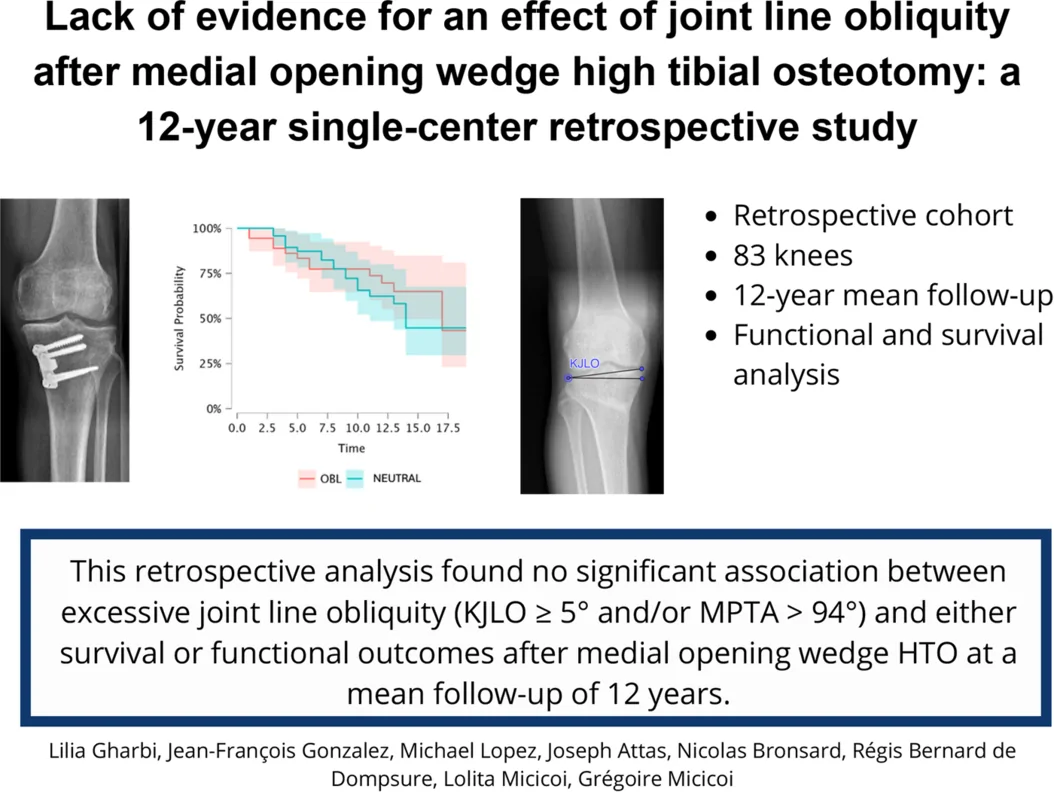

**Abbreviations**
CI: confidence interval
HKA: hip–knee–ankle angle
HR: hazard ratio
HTO: high tibial osteotomy
IKS: International Knee Society Score
JLCA: joint line convergence angle
JLO: joint line obliquity
KJLO: knee joint line obliquity
KOOS: Knee injury and Osteoarthritis Outcome Score
LDFA: lateral distal femoral angle
MPTA: medial proximal tibial angle
SD: standard deviation
SKV: Subjective Knee Value
TKA: total knee arthroplasty

## INTRODUCTION

High tibial osteotomy (HTO) is an effective option for young and active patients with medial femorotibial osteoarthritis due to varus deformity. Correction of the overall varus deformity may, however, result in tibial overcorrection and excessive knee joint line obliquity (KJLO). According to the European Society of Sport Traumatology, Knee Surgery and Arthroscopy (ESSKA) consensus, a double osteotomy should be considered when isolated tibial or femoral correction leads to a tibial deformity >94° and/or a KJLO > 5° [8]. Nevertheless, Ollivier et al. recently demonstrated that these thresholds are frequently exceeded in clinical practice [26].

The potential consequences of excessive JLO remain debated. Biomechanical data from Nakayama et al. suggested that KJLO > 5° increases stress on tibial cartilage [24]. Clinically, some authors reported better survival or functional outcomes when joint line orientation was preserved, such as Babis et al., who observed a 96% 8‐year survival after double osteotomy maintaining a neutral joint line [4], or Akamatsu et al., who associated medial proximal tibial angle (MPTA) > 95° with poorer outcomes [2]. Similarly, Song et al. found poorer radiological results with KJLO > 6° [32]. Despite this, Goshima et al. found that an MPTA > 95° did not negatively affect functional outcomes after HTO [10].

To date, most studies on JLO after HTO have focused on short‐term outcomes, with inconsistent results. In contrast, data concerning osteotomy survival in relation to KJLO are still rarely analysed at long‐term follow‐up.

The primary objective of this study was to evaluate the impact of KJLO and MPTA on functional outcomes and long‐term survival. The secondary objective was to identify risk factors for failure of HTO. This study hypothesized that the presence of an oblique joint line (KJLO > 5° and/or MPTA > 94°) would be associated with poorer functional outcomes and survival after HTO at long‐term follow‐up.

## METHODS

### Patients

This study was designed as a single‐centre retrospective cohort study conducted in accordance with the ethical standards of the 1964 Declaration of Helsinki and its later amendments. Institutional review board approval was obtained (IRB00014528_2025_03), and informed consent was obtained from all patients. All patients who underwent medial opening wedge HTO between October 2005 and December 2019 were retrospectively reviewed to ensure a minimum follow‐up of 5 years. A total of 175 valgus opening wedge high tibial osteotomies were performed during the inclusion period. Patients were eligible if they had undergone a valgus‐producing medial opening wedge HTO for symptomatic medial compartment osteoarthritis associated with a global varus deformity of the lower limb. Only procedures performed for degenerative varus osteoarthritis with complete radiographic and clinical data available at a minimum of 5 years postoperatively were included. Patients were excluded if they had undergone a distal femoral osteotomy, a double‐level osteotomy, or if the indication for HTO was post‐traumatic or related to ligamentous instability. Patients lost to follow‐up or deceased for whom outcome data were unavailable at a minimum 5‐year follow‐up were also excluded.

**Figure 1:**
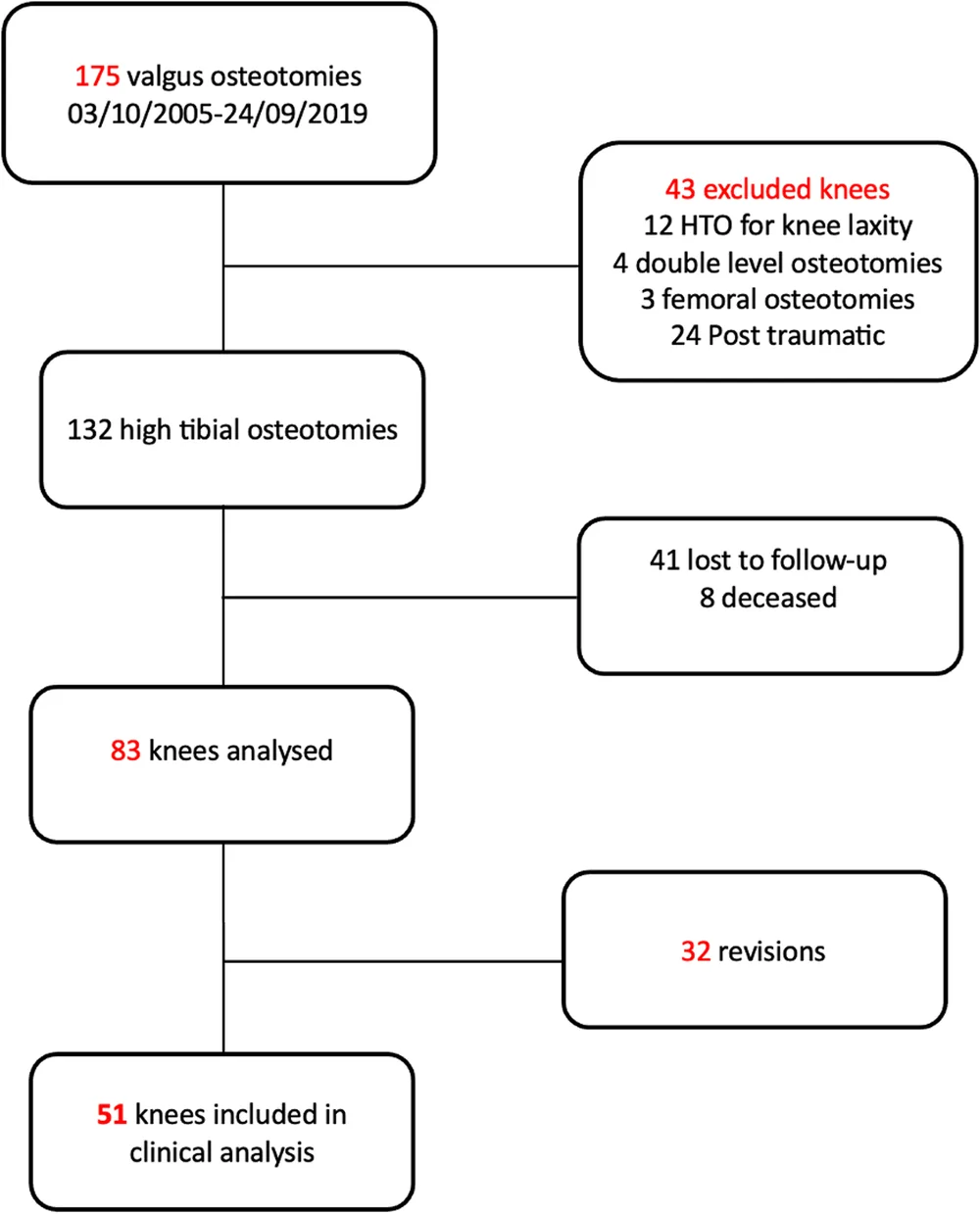
Study flowchart. HTO, high tibial osteotomy.

This study included 83 knees for survival analysis and 51 knees for functional analysis at a minimum follow‐up of 5 years (Figure 1).

The mean follow‐up was 12.3 ± 3.3 years (5–19). The following variables were collected: preoperative osteoarthritis grade, postoperative alignment, sex, age, body mass index (BMI) and smoking status.

### Surgical technique

**Figure 2:**
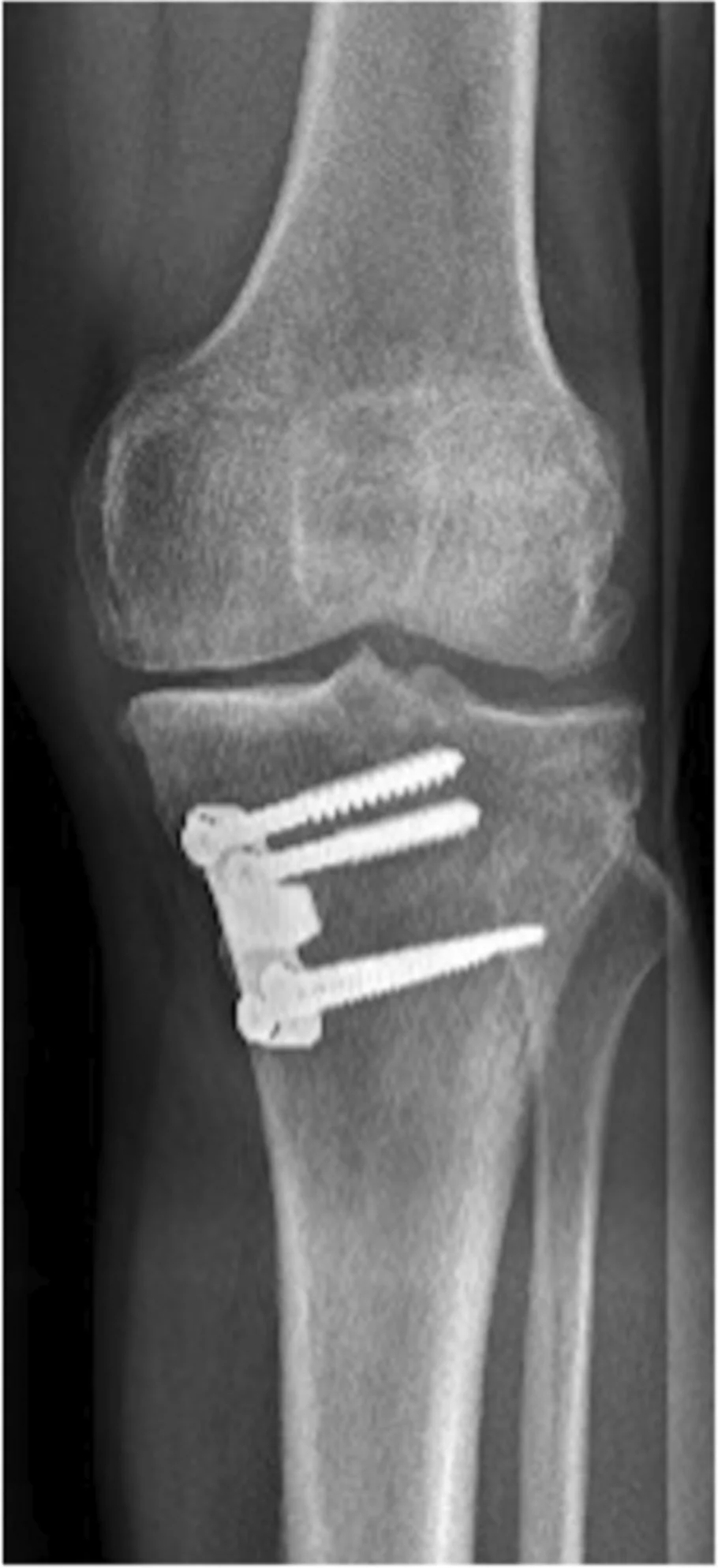
Postoperative radiograph of a high tibial osteotomy fixed with a Puddu plate at 8‐year follow‐up.

All medial opening wedge HTOs were performed for symptomatic medial compartment osteoarthritis with varus deformity in active patients. Preoperative planning was based on weight‐bearing long‐leg radiographs, targeting a valgus overcorrection of 3° [13], using either the 1 mm = 1° equivalence method [30] or the Miniaci technique [23]. The osteotomy was performed through a medial approach and fixed with a Puddu plate (Arthrex) in 63 cases (76%), an Elix plate (Biotech Ortho) in eight cases (10%), a Newclip locked plate (France) in six cases (7%), an LCP plate (DePuy Synthes) in three cases (4%) and a Numelock plate (Stryker) in two cases (3%); in one case, no plate was used (Figure 2). In all procedures, an allogenic bone substitute (Osteopure; O.S.T. Laboratories) filled the osteotomy gap. Postoperatively, patients were kept non‐weight bearing for 45 days with prophylactic anticoagulation, and progressive range of motion was initiated, targeting 90° of flexion at 1 month. Physiotherapy was initiated in the early postoperative period to maintain quadriceps activation, prevent stiffness and progressively restore range of motion and gait.

### Radiographic evaluation

For each patient, a weight‐bearing full‐leg radiograph was obtained preoperatively and within 6 months following the osteotomy. The following parameters were measured: HKA (hip–knee–ankle angle), MPTA (medial proximal tibial angle), LDFA (lateral distal femoral angle), KJLO (knee joint line obliquity) and JLCA (joint line convergence angle) [22].

The HKA was defined as the angle between the mechanical axes of the femur and tibia. The LDFA was measured between the distal femoral condylar line and the femoral mechanical axis, and the MPTA between the tibial mechanical axis and the tibial plateau line.

**Figure 3:**
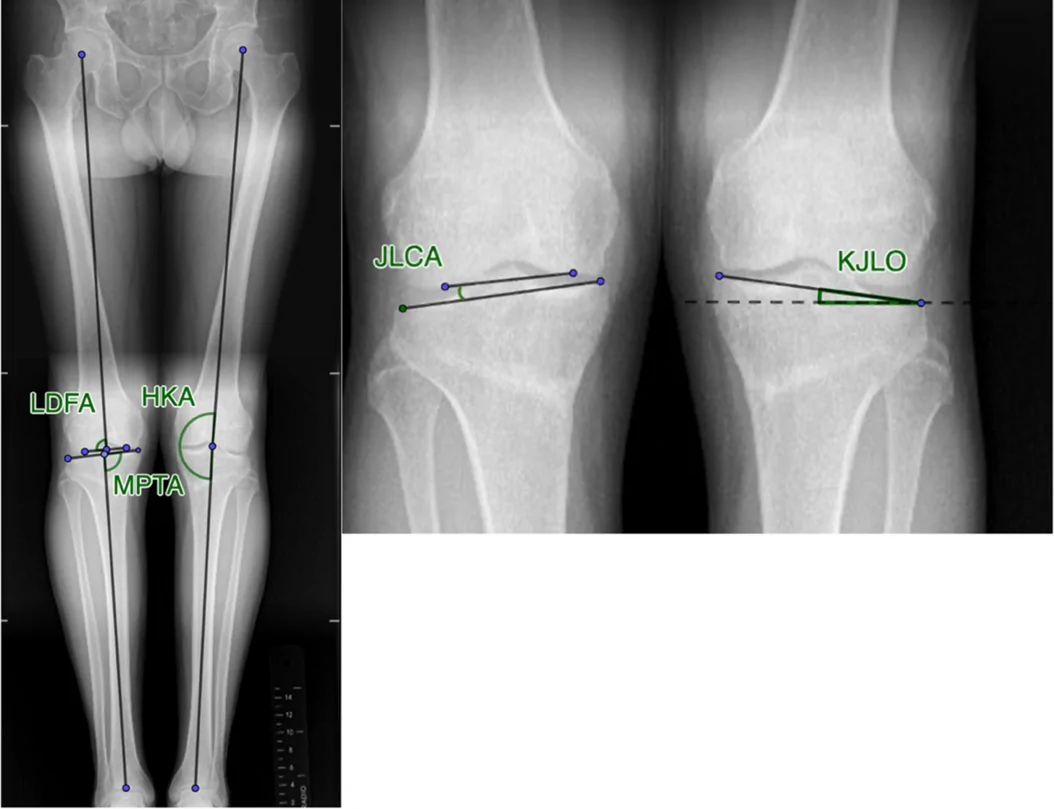
Radiographic measurements. HKA, hip–knee–ankle angle; JLCA, joint line convergence angle; KJLO, knee joint line obliquity; LDFA, lateral distal femoral angle; MPTA, medial proximal tibial angle.

The KJLO was defined as the angle between the tibial plateau and the horizontal ground line. The JLCA was measured between the distal femoral condylar line and the tibial plateau line (Figure 3). KJLO and JLCA were considered positive in cases of varus and negative in cases of valgus.

**Figure 4:**
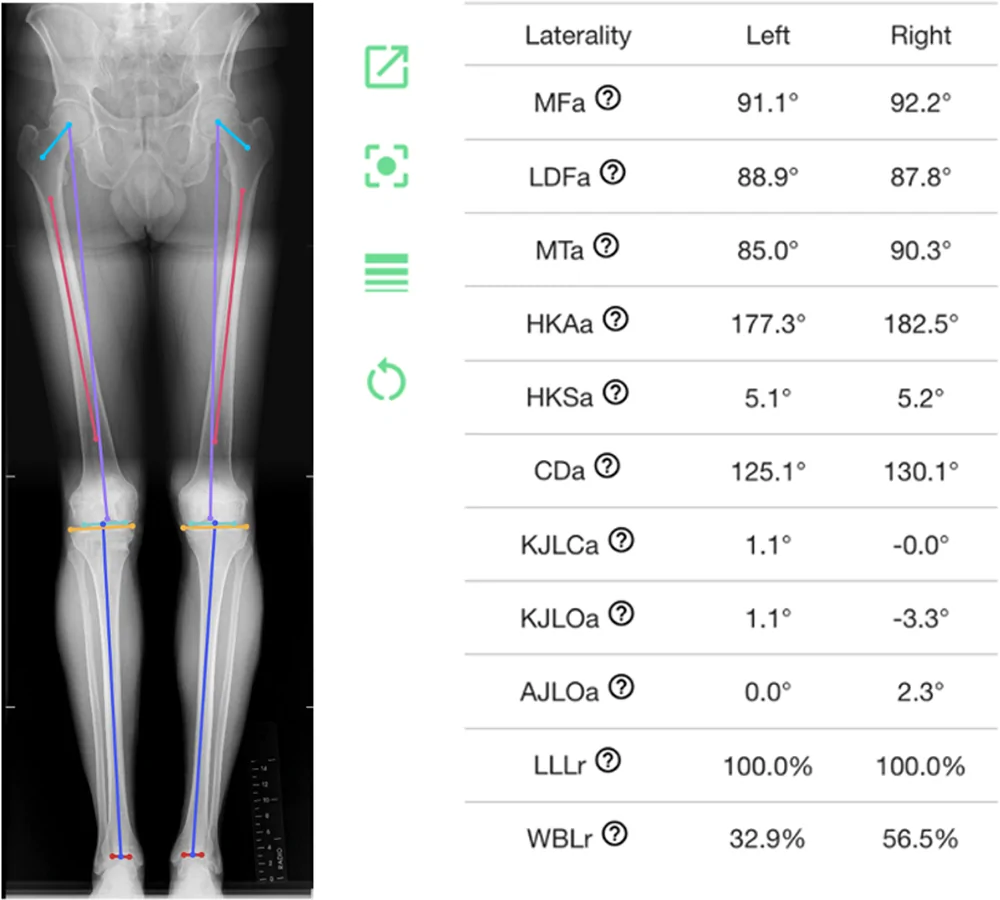
Semi‐automated angular measurements performed using the DEEMEA artificial intelligence software. The following measurements were used: HKA, hip–knee–ankle angle; JLCA, joint line convergence angle; KJLO, knee joint line obliquity; LDFA, lateral distal femoral angle; MPTA, medial proximal tibial angle.

Preoperative osteoarthritis severity was assessed according to the Ahlbäck classification [1]. Measurements were performed using a validated semi‐automated angular analysis software (Deemea; https://deemea.com), whose reproducibility and reliability have been confirmed in previous studies [6] (Figure 4).

Interobserver reproducibility was assessed by a second observer (L. G.), and intraobserver reproducibility was evaluated by repeating all measurements two weeks later by the same observer.

### Functional outcome evaluation

The following scores were collected at final follow‐up: the Knee Injury and Osteoarthritis Outcome Score (KOOS), the Subjective Knee Value (SKV) and the International Knee Society (IKS) score. The KOOS is a patient‐reported outcome ranging from 0% to 100% [28]. It includes five subscales: pain, symptoms, activities of daily living (ADL), sport and recreation function and knee‐related quality of life (QOL). The SKV was obtained by asking patients to rate their knee function as a percentage of normal, from 0 to 100 [21]. The IKS score includes two components: a subjective function subscore (IKS function) and an objective clinical score (IKS knee), each ranging from 0 to 100 points [31]. The validated French versions of the KOOS and IKS were used [16, 27].

### Statistical analysis

All statistical analyses were performed using JASP software version 0.19.3 (Amsterdam). Results were considered statistically significant at *p* < 0.050. Sample size calculation was based on the Minimal Clinically Important Difference (MCID) of 15 points on the KOOS scale, as reported by Jacquet et al. [15]. Assuming a standardized effect size of *d* ≈ 0.71, a one‐sided test, an alpha risk of 0.05 and a power of 80%, the minimum required number of participants was 50 patients in the overall cohort. Patient characteristics, functional scores and angular measurements were described using descriptive statistics, expressed as means and standard deviations or as frequencies and percentages.

Changes in radiographic angles between pre‐ and postoperative assessments were analysed using paired *t* tests. Normal distribution of continuous variables and homoscedasticity were confirmed using the Shapiro–Wilk and Levene's tests, respectively. An independent Student's *t* test was then performed to compare functional scores and angular measurements between groups. A one‐way analysis of variance (ANOVA) was used for subgroup analyses. Pearson's correlation test was used to assess the relationship between postoperative angular values and functional outcomes.

Survivorship of the high tibial osteotomies was evaluated using Kaplan–Meier survival analysis, with group comparisons performed using the log‐rank test. Revision‐free survival was defined as the absence of failure during the follow‐up period. Failures included the need for a second osteotomy or conversion to total knee arthroplasty (TKA). Removal of the osteotomy hardware was not considered a failure. Patients with incomplete follow‐up or those lost to follow‐up were considered censored at the date of their last clinical evaluation, provided that no failure had occurred before that date. Cox proportional hazards regression was performed to identify risk factors associated with osteotomy failure, both in univariate and multivariate analyses and to calculate the corresponding hazard ratios (HRs). Although the sample size was sufficient for the primary analysis based on the KOOS MCID, the study was underpowered for subgroup comparisons and Cox regression analyses, given the limited number of events and subgroup sizes.

## RESULTS

A total of 83 knees were included in the survival analysis and 51 knees in the clinical evaluation, as shown in Figure 1. The study population comprised 46 men (55%) and 37 women (45%), with a mean age of 54.1 ± 6.4 years (23–66). The mean BMI was 28.4 ± 4.0 kg/m^2^ (20.8–43.2). According to the Ahlbäck classification, 12 patients (14%) had Grade I osteoarthritis, 63 (76%) Grade II and 8 (10%) Grade III.

The right knee was operated on in 43 cases (52%) and the left in 40 (48%). The mean opening wedge correction was 10.4 ± 1.9 mm (5–15). Previous surgeries included partial medial meniscectomy in 16 cases (19%) and anterior cruciate ligament (ACL) reconstruction in 3 cases (4%). Concomitant procedures performed during HTO included lateral retinacular release in 5 patients (6%) and lateral capsular release in 9 patients (11%). A hinge fracture was identified in 12 cases (14%) as Grade I and in 1 case (1%) as Grade III, according to the Takeuchi classification [33].

Baseline patient characteristics are summarized in Table 1.

**Table 1:** Demographic characteristics of the study population.

Characteristics	Population, *n* = 83
Gender	37 women (45%)
	46 men (55%)
Age at surgery	54.1 ± 6.4 (23–66)
Osteoarthritis stage (Ahlbäck classification)	
I	12 (14%)
II	63 (76%)
III	8 (10%)
BMI	28.4 ± 4 (20.8–43.2)
Side	
Right	43 (52%)
Left	40 (48%)
Opening wedge correction (millimetres)	10.4 ± 1.9 (5–15)
Postoperative alignment	
HKA < 180°	23 (28%)
HKA 180°–183°	43 (52%)
HKA > 183°	17 (20%)

Abbreviations: BMI, body mass index in kg/m^2^; HKA, hip–knee–ankle angle.

### Analysis of angular corrections

Intraobserver agreement was good (correlation coefficient < 0.9) for JLCA and KJLO, and excellent (>0.9) for all other angular parameters (Table 2).

**Table 2:** Inter‐ and intra‐observer reliability of angular parameters.

Variable	Intra‐observer	Inter‐observer
HKA	0.99 (0.99–1.00)	0.99 (0.98–1.00)
MPTA	0.90 (0.80–0.96)	0.98 (0.95–0.99)
LDFA	0.98 (0.96–0.99)	0.94 (0.89–0.95)
JLCA	0.87 (0.74–0.94)	0.96 (0.93–0.98)
KJLO	0.82 (0.64–0.91)	0.96 (0.94–0.98)

*Note*: ICC > 0.9: excellent correlation; ICC > 0.75: good correlation; ICC < 0.75: moderate correlation.

Abbreviations: HKA, hip–knee–ankle angle; ICC, Intraclass Correlation Coefficient; JLCA, joint line convergence angle; KJLO, knee joint line obliquity; LDFA, lateral distal femoral angle; MPTA, medial proximal tibial angle.

The HKA, MPTA and JLCA angles were significantly increased postoperatively (*Δ* = 7.8° ± 12.2; *Δ* = 6.1° ± 11.8; and *Δ* = 0.3° ± 1.6, respectively; *p* < 0.01) (Table 3).

**Table 3:** Comparison of radiographic parameters before and after valgus high tibial osteotomy.

	Preoperative	*Δ*	Postoperative	*p* Value
Radiographic outcomes				
HKA (°)	174 ± 3.3 (165.0–178.2)	8°	181.8 ± 3.0 (176.5–192.8)	<0.010
MPTA (°)	86.1 ± 2.9 (77.1–92.0)	5.9°	92.2 ± 4.0 (82.6–99.2)	<0.010
LDFA (°)	89.8 ± 2.4 (82.7–96.3)	0.4°	89.4 ± 2.3 (83.6–95.3)	0.932
JLCA (°)	−2.1 ± 2.2 (−8.0 to 2.7)	1.9°	−1.2 ± 2.5 (−8.5 to 4.5)	<0.001
KJLO (°)	−0.9 ± 3.0 (−6.5 to 5.7)	2.25°	−2.1 ± 3.8 (−8.4 to 9.0)	<0.001

*Note*: Results are presented as mean ± standard deviation (minimum–maximum).

Abbreviations: HKA, hip–knee–ankle angle; JLCA, joint line convergence angle; KJLO, knee joint line obliquity; LDFA, lateral distal femoral angle; MPTA, medial proximal tibial angle.

The KJLO angle was significantly decreased postoperatively (*Δ* = –1.2° ± 2.2; *p* < 0.01). The LDFA angle remained unchanged. Postoperatively, 23 patients (28%) were undercorrected (HKA < 180°), 43 patients (52%) were within target alignment (HKA = 180°–183°) and 17 patients (20%) were overcorrected (HKA > 183°).

### Survival and functional outcome analysis

**Figure 5:**
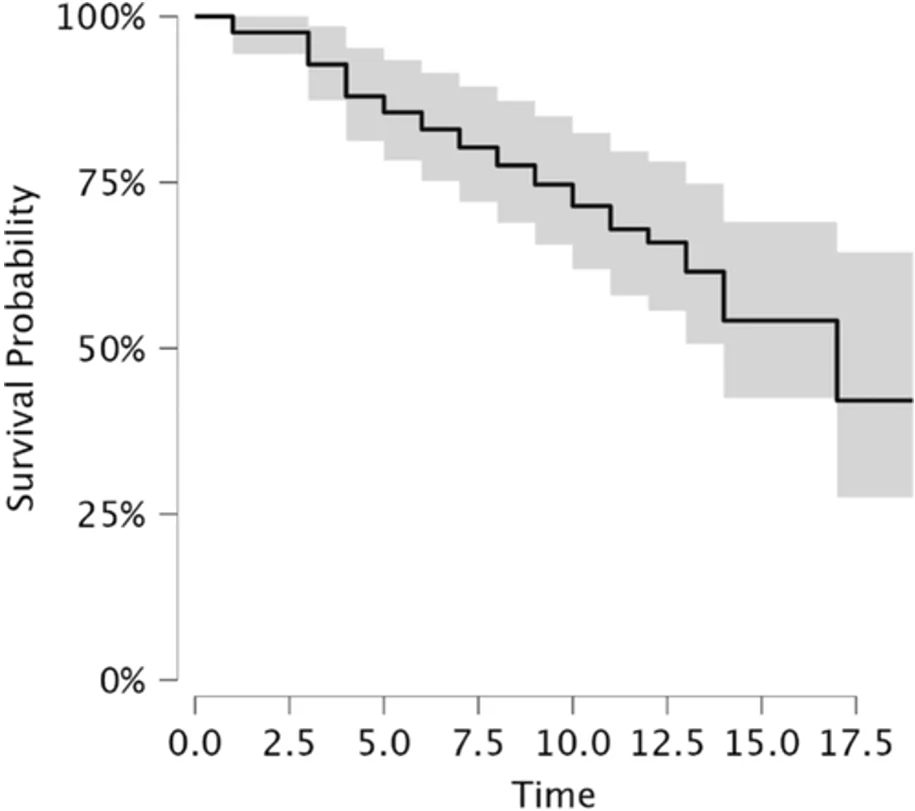
Kaplan–Meier survival curve after high tibial osteotomy. Mean survival was 13.9 ± 0.7 years. Survival rates were 85.5% at 5 years, 71.4% at 10 years and an estimated 54.2% at 15 years.

At final follow‐up, 32 knees had undergone conversion to TKA. The mean time to conversion was 13.9 ± 0.7 years; overall survival, as determined by Kaplan–Meier analysis, was 85.5% at 5 years and 71.4% at 10 years (Figure 5).

Among patients who had not undergone arthroplasty at final follow‐up, the mean KOOS was 61 ± 23, the SKV was 64% and the IKS scores were 84.3 ± 10.4 for the IKS Knee Score and 63.2 ± 23 for the IKS Function Score.

No significant correlation was found between postoperative angular parameters (HKA, MPTA, JLCA and KJLO) and functional scores (KOOS, IKS Knee and IKS Function) according to Pearson analysis (*p* > 0.050).

### Influence of JLO on functional and survival outcomes

Postoperatively, 29 patients (35%) had an MPTA > 94°, and 29 patients (35%) had a KJLO > 5°, resulting in 36 patients (44%) meeting the ESSKA definition of excessive JLO (MPTA > 94° and/or KJLO > 5°).

The two groups (with or without JLO) were comparable in terms of age (*p* = 0.68), osteoarthritis grade (*p* = 0.33), BMI (*p* = 0.84), presence of a hinge fracture (*p* = 0.69) and smoking status (*p* = 0.82). There were significantly more males in the neutral joint line group than females (66% vs. 44%; *p* = 0.03) (Table 4).

**Table 4:** Comparison of demographic characteristics between the ‘oblique joint line’ and ‘neutral joint line’ groups.

	Oblique joint line (*n* = 36)	Neutral joint line (*n* = 47)	*p*
Smoking status	4 (11%)	6 (13%)	0.818
Age at surgery	55.3 ± 8	53.2 ± 8.5	0.681
Per‐operative fracture	5 (14%)	8 (17%)	0.691
Preoperative osteoarthritis stage ( > 2)	2 (5%)	6 (13%)	0.329
Coronal alignment (HKA < 180 or >183°)	22 (61%)	18 (38%)	0.310
Degree of correction	10.7 ± 2.4	9.8 ± 2.2	0.121
BMI	28.7 ± 5.2	28.9 ± 5.3	0.840
Male sex	16 (44%)	31 (66%)	0.031

Abbreviations: BMI, body mass index in kg/m^2^; HKA, hip–knee–ankle angle.

Functional outcomes were similar between the two groups, oblique or neutral joint line; for the KOOS (respectively, 59.1 ± 18.1 vs. 62.2 ± 19.3; *p* = 0.632), the SKV (respectively, 63.2 ± 21.8 vs. 64.8 ± 19.9; *p* = 0.78), the IKS Knee Score (respectively, 84 ± 11.8 vs. 83.6 ± 9.3; *p* = 0.89) and the IKS Function Score (respectively, 62.9 ± 21.8 vs. 61.9 ± 24.5; *p* = 0.89).

ANOVA analysis showed no effect of sex (*R* ^2^ = 0.01; *p* = 0.48), age (*R* ^2^ = 0.02; *p* = 0.75), BMI (*R* ^2^ = 0.01; *p* = 0.44), smoking status (*R* ^2^ = 0.03; *p* = 0.28) or osteoarthritis stage (*R* ^2^ = 0.01; *p* = 0.52) on functional scores.

**Figure 6:**
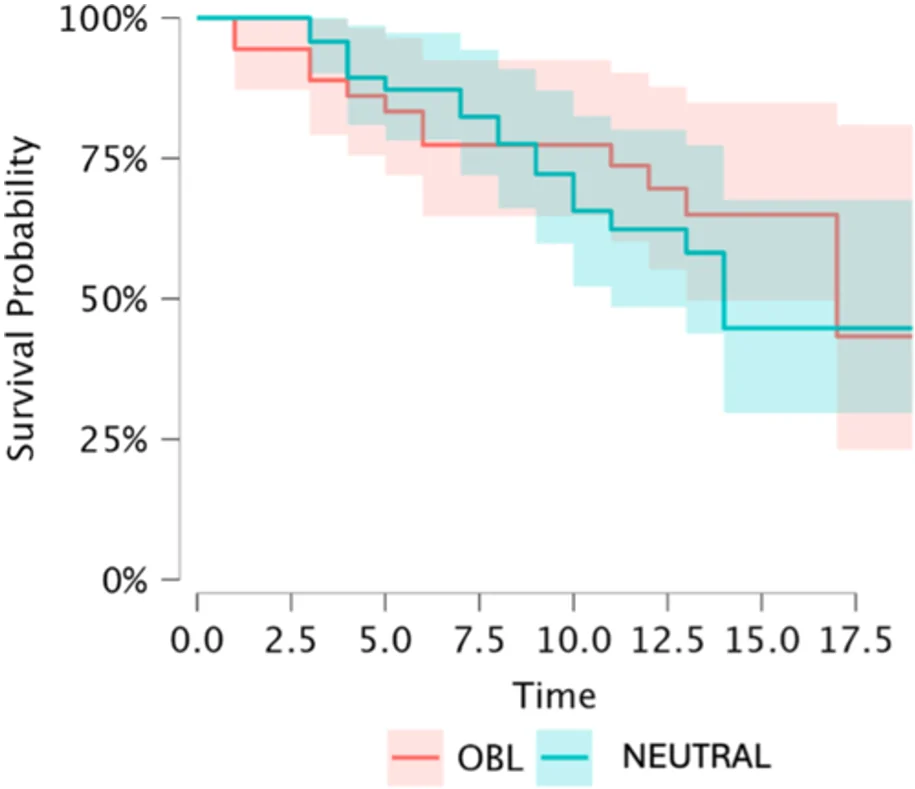
Kaplan–Meier survival curve comparing the oblique joint line group (KJLO > 5° and/or MPTA > 94°) and the neutral joint line group. Mean revision‐free survival was comparable between the oblique joint line group (14.2 ± 1.1 years) and the neutral joint line group (13.6 ± 0.9 years) (*p* > 0.050). KJLO, knee joint line obliquity; MPTA, medial proximal tibial angle; OBL, oblique.

Univariate and multivariate Cox regression analyses did not show a significant influence of JLO (MPTA > 94° and/or KJLO > 5°) on revision‐free survival (*p* > 0.050) (Figure 6).

### Analysis of risk factors for failure after HTO

In both univariate and multivariate Cox regression analyses, obesity (defined as a BMI greater than 30) and sex had no impact on revision‐free survival (*p* > 0.050).

Advanced age > 55 years was significantly associated with an increased risk of revision (HR = 1.6; *p* < 0.050), with an annual risk of 3.4%. Smoking was also associated with a higher risk of revision (HR = 1.8; *p* < 0.050), with an estimated annual risk of 7.3%.

A preoperative advanced osteoarthritis stage (Ahlbäck ≥ 3) was also associated with failure (HR = 2; *p* < 0.050), with an annual risk of 3.4% for Grade III osteoarthritis. Finally, a postoperative alignment outside the target (HKA < 180° or HKA > 183°) was also associated with a higher risk of conversion to TKA (HR = 2.2; *p* < 0.050) (Tables 5 and 6).

**Table 5:** Univariate Cox regression analysis of factors influencing survival after high tibial osteotomy.

Variable	Mean survival	HR (95% CI)	*p* value	
Joint line			
Oblique	14.2	1.28 (0.6–2.6)	0.488	
Neutral	13.6			
Postoperative HKA				
180°–183°	15.8	2.5 (1.2–5.2)	0.022	
<180° ou > 183°	12.3			
Preoperative osteoarthritis stage				
Ahlbäck 1 or 2	14.3	3 (1.1–8)	0.034	
Ahlbäck 3	8.7			
Gender				
Woman	15.2	1.7 (0.8–3.5)	0.127	
Man	13			
Age at surgery				
<55 years	15.6	1.9 (0.9–3.6)	0.029	
>55 years	12.8			
BMI				
<30	15.3	1.8 (1–3.7)	0.394	
>30	13.2			
Smoking status				
Yes	14.4	2.3 (0.9–5.5)	0.028	
No	10.6			

Abbreviations: BMI, body mass index in kg/m^2^; CI, confidence interval; HKA, hip–knee–ankle angle; HR, hazard ratio.

**Table 6:** Multivariate Cox regression analysis of factors influencing survival after high tibial osteotomy.

Variable	Mean survival	HR (95% CI)	*p* value
Postoperative HKA			
180°–183°	15.8	2.2 (1–4.9)	0.046
<180° or > 183°	12.3		
Preoperative osteoarthritis stage			
Ahlbäck 1 or 2	14.3	2 (0.7–5.8)	0.041
Ahlbäck 3	8.7		
Age at surgery			
<55 years	15.6	1.6 (0.8–3.3)	0.048
>55 years	12.8		
Smoking status			
Yes	14.4	1.8 (0.7–4.7)	0.045
No	10.6		

Abbreviations: BMI, body mass index in kg/m^2^; CI, confidence interval; HKA, hip–knee–ankle angle; HR, hazard ratio.

## DISCUSSION

The primary finding of this study is that postoperative excessive JLO (KJLO > 5° and/or MPTA > 94°) after medial opening‐wedge HTO did not affect functional outcomes or survival at a mean follow‐up of 12 years.

The results of this study support the long‐term efficacy of medial opening‐wedge HTO in the management of medial femorotibial osteoarthritis in young and active patients. The overall survival observed in the present cohort was 85.5% at 5 years and 71.4% at 10 years. These findings are consistent with those reported in the literature. Niinimäki et al. reported a 5‐year survival rate of 89% and a 10‐year survival rate of 73% in a cohort of 3195 knees [25]. Similarly, the cohort reported by van Wulfften Palthe et al. showed a 10‐year survival rate of 75% and a 15‐year survival rate of 55% [35].

The impact of JLO remains a matter of debate. In the present series, the KJLO and MPTA thresholds recommended by ESSKA were not respected in 44% of cases, a proportion similar to the rates reported by Ollivier et al., who found an MPTA > 94° in 41% of cases and a KJLO > 5° in 29.4% of cases [26]. Nevertheless, despite this high frequency, no significant difference in functional scores or survival was observed between patients with an oblique joint line and those with a neutral one. These findings align with those of several authors. For instance, Rosso et al. conducted a study of 92 patients with a mean follow‐up of 10 years, finding no significant difference in IKS scores [29]. Likewise, Lee et al. reported no significant difference in WOMAC or SF‐36 scores at 1‐year follow‐up [19]. In contrast, some studies have reported opposing results. Akamatsu et al. found significantly lower KSS and KOOS scores in a series of 86 patients with JLO (2). Similarly, Kim et al. demonstrated a significant decrease in KSS and SF‐36 scores in patients with JLO at a minimum follow‐up of 4 years [18]. These discrepancies may be attributed to methodological differences, variations in inclusion criteria or inconsistent definitions of JLO.

It should also be noted that the mean functional outcomes in this cohort were relatively modest (KOOS, IKS Function and SKV), likely reflecting progressive arthritic changes in many patients who have not yet undergone total knee replacement. However, these results are comparable to those reported by Hantes et al., who found similar KOOS (69.4 ± 14) scores at long‐term follow‐up after medial opening‐wedge HTO [12]. This finding highlights that, despite satisfactory survival, long‐term functional recovery after HTO may remain incomplete.

Additionally, Cox regression analysis identified several risk factors associated with HTO failure. Smoking was a significant risk factor, with an HR of 1.8. Although no study has clearly assessed the impact of smoking on HTO survival, a recent meta‐analysis by Al Musabi et al. showed that smokers had a twofold increased risk of non‐union following HTO, which may indirectly compromise surgical outcomes [3].

Older age also emerged as an unfavourable prognostic factor. Patients over 55 years of age had a significantly higher risk of conversion to arthroplasty (HR = 1.6), consistent with findings by Bonasia et al., who reported a fivefold increase in failure risk beyond the age of 56 [5]. Moreover, Howells et al. demonstrated that patients under 55 years of age had better long‐term survival after HTO [14].

The severity of osteoarthritis at the time of surgery also played a key prognostic role. In the present series, advanced osteoarthritis (Ahlbäck ≥ 3) was associated with a higher risk of conversion to arthroplasty (HR = 2.0). This finding is consistent with the work by Efe et al., who identified a Kellgren–Lawrence grade of greater than 2 as a predictor of failure [7]. Thus, in older patients or those with advanced osteoarthritis, the survival time of the osteotomy is generally shorter. Nevertheless, some of these patients may remain active or reluctant to undergo arthroplasty, and a joint‐preserving strategy can provide a valuable option to delay joint replacement for several years. Sex was not found to be a risk factor in this study, although the literature remains divided on this point. Raaij et al. suggested that female sex was a risk factor [34], whereas Gstöttner et al. found no difference according to sex [11].

Postoperative alignment outside the target range, defined as HKA < 180° or > 183°, was also associated with an increased risk of conversion to TKA (HR = 2.2). This finding has already been highlighted in the literature: Jin et al. reported an association between undercorrection (< 180°) and failure, while Hernigou et al. showed that overcorrection into valgus (> 186°) or failure to achieve slight valgus overcorrection (< 183°) increased the risk of arthritic progression in the lateral and medial compartments, respectively [13, 17]. Lastly, obesity did not affect survival in this study, in contrast to other publications that reported a negative impact of BMI > 30 on HTO outcomes [9]. However, Mabrouk et al. also found no significant effect of BMI on survival [20].

This study has some limitations. Its retrospective and single‐centre design may limit the generalizability of the findings; however, the strict inclusion criteria provided a homogeneous cohort with consistent indications and surgical technique. Although angular parameters were assessed using standardized long‐leg radiographs, measurement bias may persist due to positioning variability. This was particularly relevant for KJLO, for which the intraobserver ICC was only moderate‐to‐good (0.82); nevertheless, interobserver reproducibility remained excellent (0.96), supporting the reliability of the measurements.

The functional analysis included only 51 patients, which represents a borderline sample size; however, this number fulfilled the a priori sample size requirement, and all eligible patients with complete data were included. Extrapolation of these findings is therefore limited by the relatively small number of patients included in this study. Similarly, Cox regression analyses were limited by the relatively small number of events, making the results statistically fragile; yet, the risk factors identified were consistent with those reported in larger studies, which supports the external validity of the present findings. Comparisons between neutral and oblique joint line groups may have been underpowered, and a Type II error cannot be excluded; nonetheless, the absence of even a trend suggests that a major effect is unlikely. Finally, although some patients were lost to follow‐up, this limitation was balanced by the long‐term observation period, with a mean follow‐up of more than 12 years, which represents one of the strengths of this study.

## CONCLUSION

This retrospective analysis found no significant association between excessive JLO (KJLO ≥ 5° and/or MPTA > 94°) and either survival or functional outcomes after medial opening wedge HTO at a mean follow‐up of 12 years. Factors negatively influencing survival after HTO were smoking, age > 55 years, advanced osteoarthritis and out‐of‐target postoperative alignment. These findings should be confirmed by larger series.

## AUTHOR CONTRIBUTIONS

**Lilia Gharbi**: Manuscript writing; data collection; statistical analysis. **Régis Bernard de Dompsure**: Assistance with protocol development and manuscript review. **Nicolas Bronsard and Jean‐François Gonzalez**: Article review. **Lolita Micicoi, Michael Lopez and Joseph Attas**: Review and submission. **Grégoire Micicoi**: Study design and revision of all versions. All authors approved the final manuscript.

## CONFLICT OF INTEREST STATEMENT

Jean‐François Gonzalez is a consultant for the Amplitude group. Nicolas Bronsard is a consultant for Si‐Bone. Régis Bernard de Dompsure is a consultant for Corin. The remaining authors declare no conflict of interest.

## ETHICS STATEMENT

This study was conducted in accordance with the Declaration of Helsinki. Institutional Review Board approval was obtained: IRB00014528_2025_03. Written informed consent was obtained from all patients included in the study.

## Data Availability

The datasets generated and analysed during the current study are available from the corresponding author on reasonable request.
